# Supplementary material for: Synthesis of (+)-(R)-Tiruchanduramine
Source: Molecules. 2022 Feb 16;27(4):1338. doi: 10.3390/molecules27041338 (PMC8880061; doi:10.3390/molecules27041338)
Supplement: Supplementary file 1 [file molecules-27-01338-s001.zip › molecules-1586570-supplementary.pdf]

Supplementary materials

Additional experimental details: Preparation of (*S*)-3.

***S*-Malic acid dimethyl ester.**[1] Acetyl chloride (17.0 mL, 0.24 mol) was carefully added to cooled (0 °C) MeOH (330 mL). *S*-(-)-Malic acid (50.3 g, 0.38 mol) was then added to the solution which was stirred to rt stirred overnight. The reaction was concentrated the residue distilled under reduced pressure (115-120 °C, 14 mm Hg) to give the dimethyl ester **2** (34.5 g, 0.21 mol) in 57 % yield. **Rf** 0.67 (EtOAc). Data was in agreement with the literature.[1]

Methyl *S*-3,4-dihydroxybutanoate.[1] Borane-DMS complex (2 M, 32.0 mL, 64.0 mmol, 1.0 equiv.) was added to a cooled (0 °C) solution of the dimethyl ester **2** (10.1 g, 63.0 mmol, 1.0 equiv.) in dry THF (125 mL) and the mixture stirred for 30 min. A catalytic amount of $NaBH_4$ (134.1 mg, 3.54 mmol, 0.06 equiv.) was then added and stirring continued for 1h. Once complete (TLC), methanol (40 mL) was added and stirring continued overnight. The mixture was purified by column chromatography (EtOAc) to give diol **3** (6.42 g) in 77% yield. **Rf** 0.30 (EtOAc). Data was in agreement with the literature.[1]

***S*-2-(2,2-Dimethyl-1,3-dioxolan-4-yl)ethan-1-ol.**[1] 2-Methoxypropene (9.0 mL, 94.0 mmol, 2.34 equiv.) was added to a stirred solution of diol **3** (5.39 g, 40.2 mmol, 1.0 equiv.) and a catalytic amount of *para*-toluenesulphonic acid (0.2 g, 1.16 mmol, 0.03 equiv.) dissolved in DCM (40 mL, 0 °C). After 1 h, TLC analysis confirmed the reaction had gone to completion and the solvent was removed by rotary evaporation to give **4** (8.66 g) which was used in the next step without further purification. Rf 0.75 (EtOAc); Data was in agreement with the literature.[1] $LiAlH_4$ (1.46 g, 39.5 mmol, 1.08 equiv.) was added in portions to a cooled (0 °C) of crude compound **4** (8.66 g) in dry Et_2O (130 mL). After 2 h TLC indicated a complete reaction and ethyl acetate (10 mL) was added and stirring continued for 1 h. A solution of NH_4Cl (aq. sat. 60 mL) was added, the organic layer separated, the aqueous layer extracted with ether (4 x 50 mL). The combined organic phases were dried ($MgSO_4$), evaporated under reduced pressure and purified by column chromatography on silica gel (40 % EtOAc in petroleum ether) to give **5** (4.34 g, 21.7 mmol in 81% yield $[\alpha]_D^{17.6}$ = +2.5 (Lit. +2.45).[1] **Rf** 0.46 (EtOAc). Further data was in agreement with the literature.[2]

***S*-4-(2-Azidoethyl)-2,2-dimethyl-1,3-dioxolane.**[2] PPh_3 (2.78 g, 10.59 mmol, 1.2 equiv.) and DIAD (2.14 g, 2.14 mmol, 2.14 equiv.) were added sequentially to a cooled (0 °C) and stirred solution of alcohol **5** (1.29 g, 8.82 mmol, 1 equiv.) in dry THF (20 mL). After 15 min, DPPA (2.28 mL, 10.59 mmol, 1.2 equiv) was then added dropwise over 15 min and stirring was continued for 24 h. After evaporation, column chromatography on silica gel (20-40% EA in PE) gave the azide **6** (1.29 g, 8.83 mmol in 85 % yield. **Rf** 0.31 (20% EA in PE) Data was in agreement with the literature.[2]

***S*-2-(2,2-dimethyl-1,3-dioxolan-4-yl)ethan-1-amine (*S*)-3.**[3] PPh_3 (2.24 g, 8.55 mmol, 1.2 equiv.) and H_2O (2.0 mL, 0.11 mol, 15.5 equiv.) was added to a solution of azide **6** (1.22 g, 7.13 mmol, 1.0 equiv.) in THF (10 mL) and the mixture stirred at rt overnight. The reaction was then evaporated to dryness and the residue dissolved in $CHCl_3$ which was then dried ($MgSO_4$), filtered and evaporated. The residue was then dissolved in a minimum of $CHCl_3$ (ca. 5 mL) and the solution diluted with diethyl ether (50 mL). The supernatant was filtered from the precipitated (TPPO) and evaporated. This process was repeated and on evaporation gave the crude amine **7** (1.41 g), which was used in the next step without further purification.

1 Gaunt, M. J.; Jessiman, A. S.; Orsini, P.; Tanner, H. R.; Hook, D.F.; Ley, S. V.; *Org. Lett.* **2003**; 5, 4819-4822
2 Taber, D. F, Deker, P. B.; Fales, H. M.; Jones, T. H.; Lloyd, H. A.; *J. Org. Chem.* **1988**; 53 2968-2971.
3 Evans, D. M.; Hughes, J.; Jones, L. F.; Murphy, P.J.; Falfushynska, H.; Horyn, O.; Sokolova, I. M.; Christensen, J.; Coles, S. J.; Rzymski. P.; *Chemosphere* **2019** 234, 139-147.

Spectroscopic information for Compounds

7 (^{1}H NMR (COSY insert) and ^{13}C NMR DEPTQ (HSQC insert) – page 3

8 (^{1}H NMR (COSY insert) and ^{13}C NMR DEPTQ (HSQC insert) – page 4

9 (^{1}H NMR (COSY insert) and ^{13}C NMR DEPTQ (HSQC insert) -page 5

14 (^{1}H NMR (COSY insert) and ^{13}C NMR DEPTQ (HSQC insert) – page 6

16 (^{1}H NMR (COSY insert) and ^{13}C NMR DEPTQ (HSQC insert) – page 7

17 (^{1}H NMR (COSY insert) and ^{13}C NMR DEPTQ (HSQC insert) – page 8

(*R*)-5 (^{1}H NMR (COSY insert) and ^{13}C NMR DEPTQ (HSQC insert) – page 9

(*+)-(R)*-Tiruchanduramine 1·HCl.
^{1}H NMR (COSY insert), ^{13}C NMR DEPTQ and HSQC – pages 10-11

***(+)-(R)*-Tiruchanduramine 1·HCO_2H.**
^{1}H NMR, COSY insert, ^{13}C NMR DEPTQ and HSQC – pages 12-13

***S*-*N*-(2-(2,2-Dimethyl-1,3-dioxolan-4-yl)ethyl)-9*H*-pyrido[3,4-*b*]indole-3-carboxamide 7**

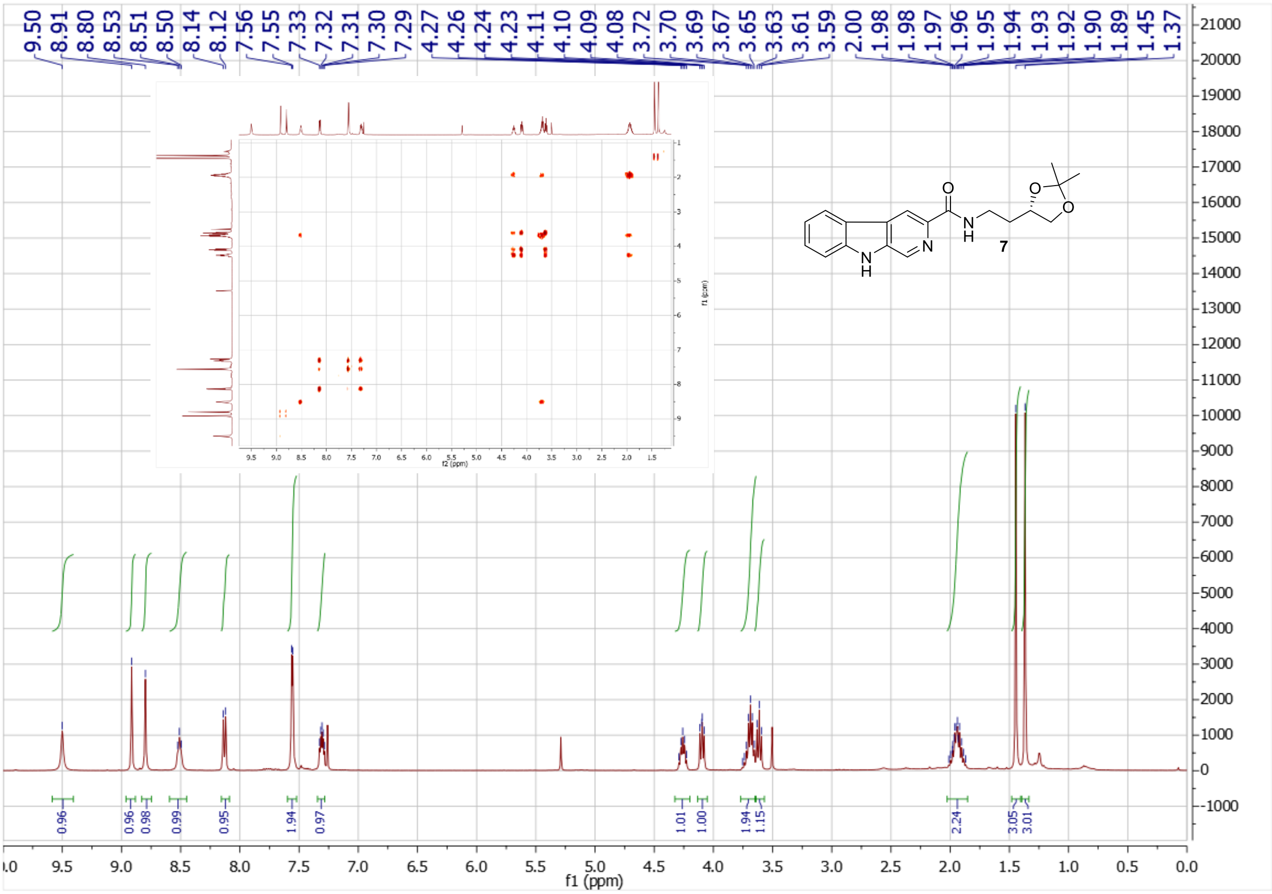

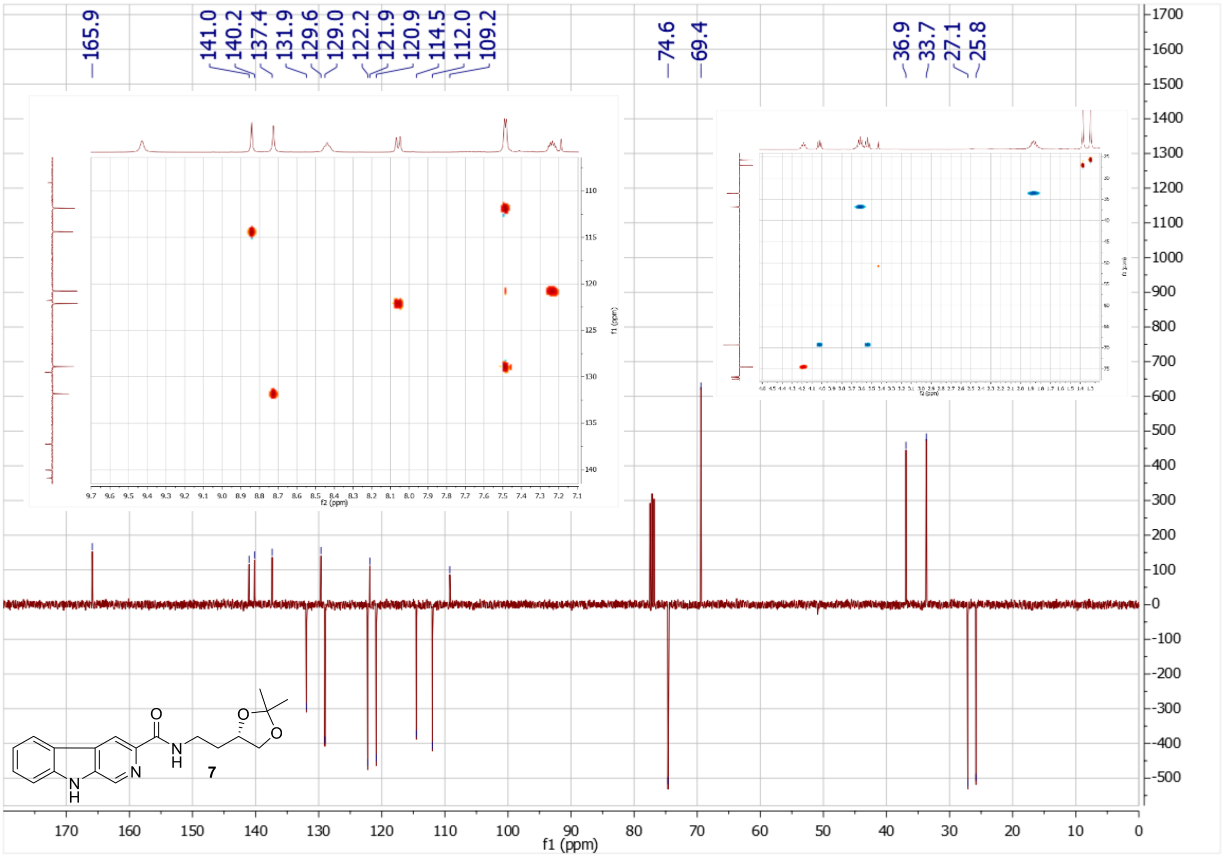

***tert*-Butyl *S*-3-((2-(2,2-dimethyl-1,3-dioxolan-4-yl)ethyl)carbamoyl)-9*H*-pyrido[3,4-*b*]indole-9-carboxylate 8**

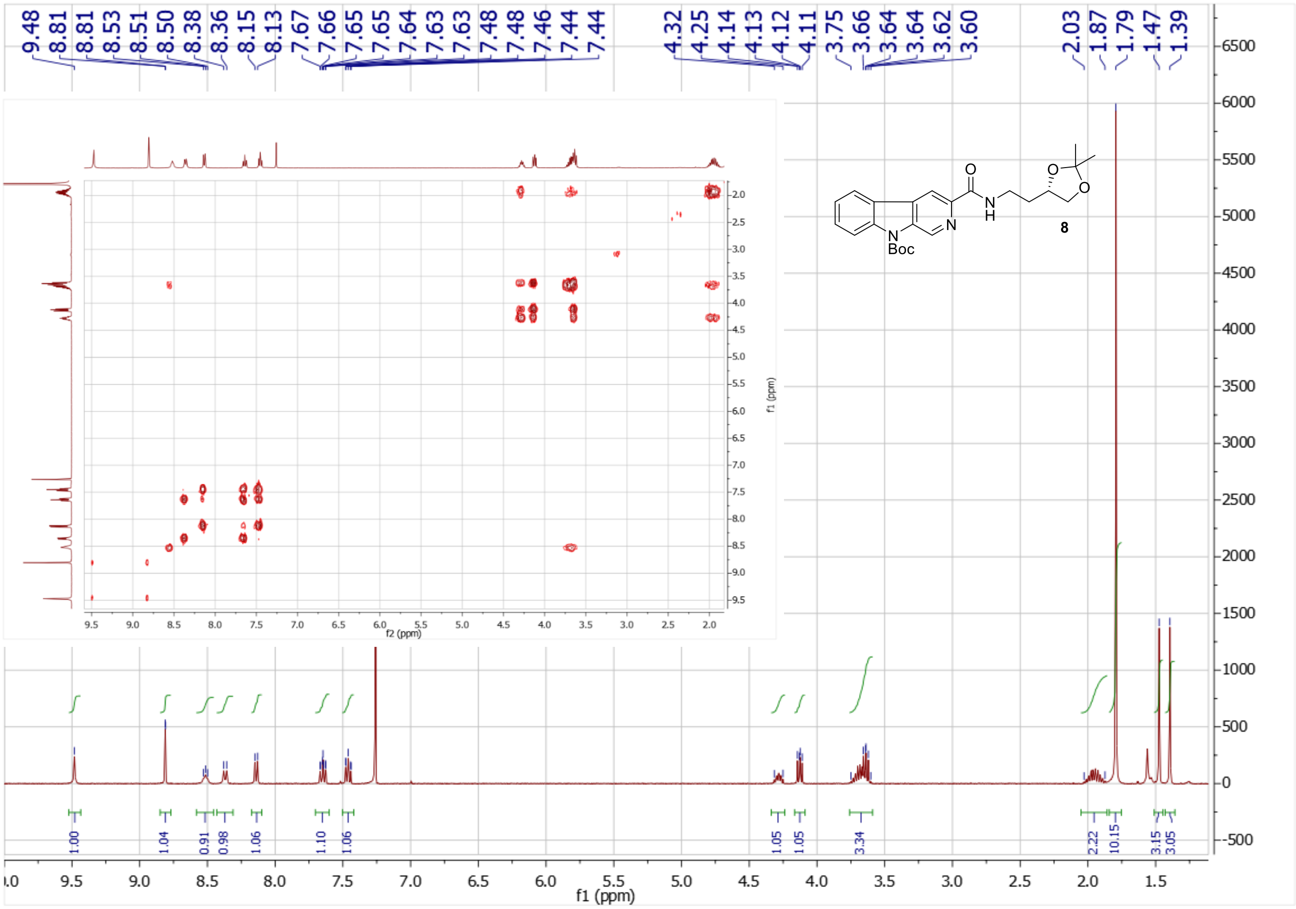

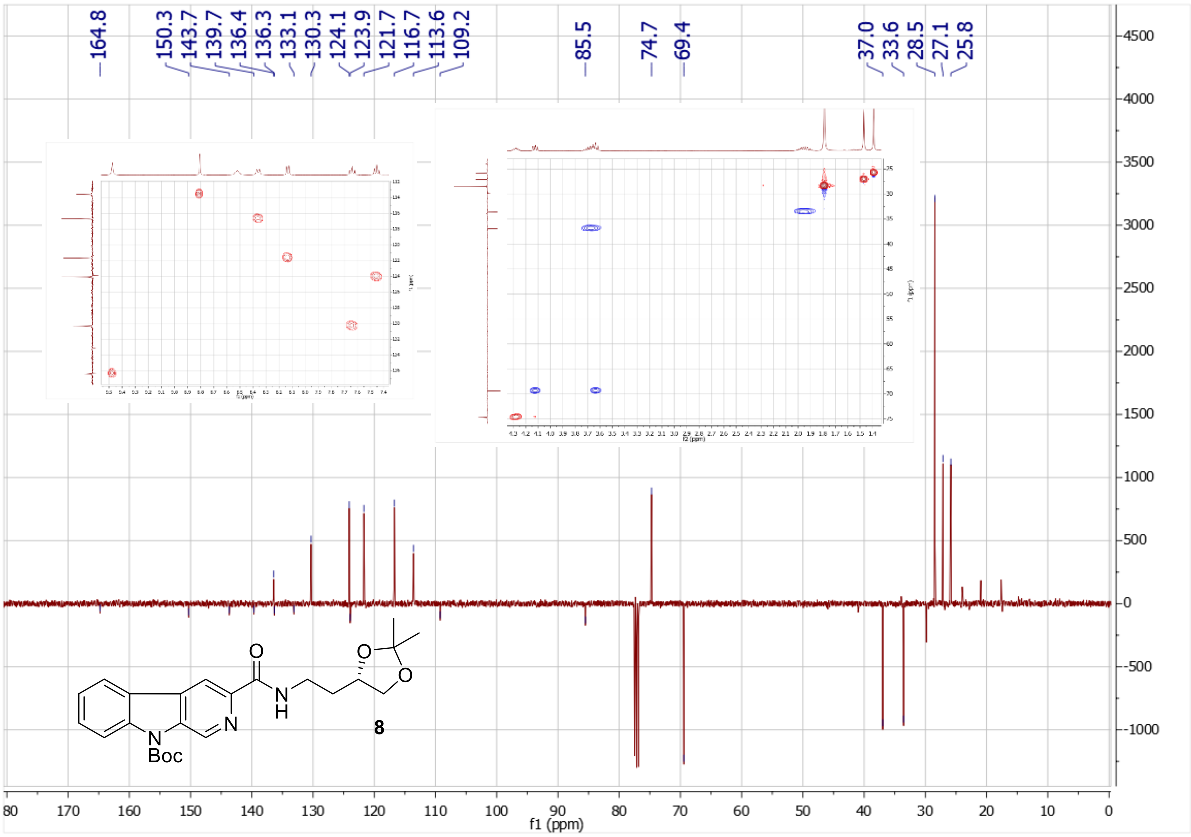

tert-Butyl (*S*)-3-((3,4-dihydroxybutyl)carbamoyl)-9H-pyrido[3,4-b]indole-9-carboxylate 9

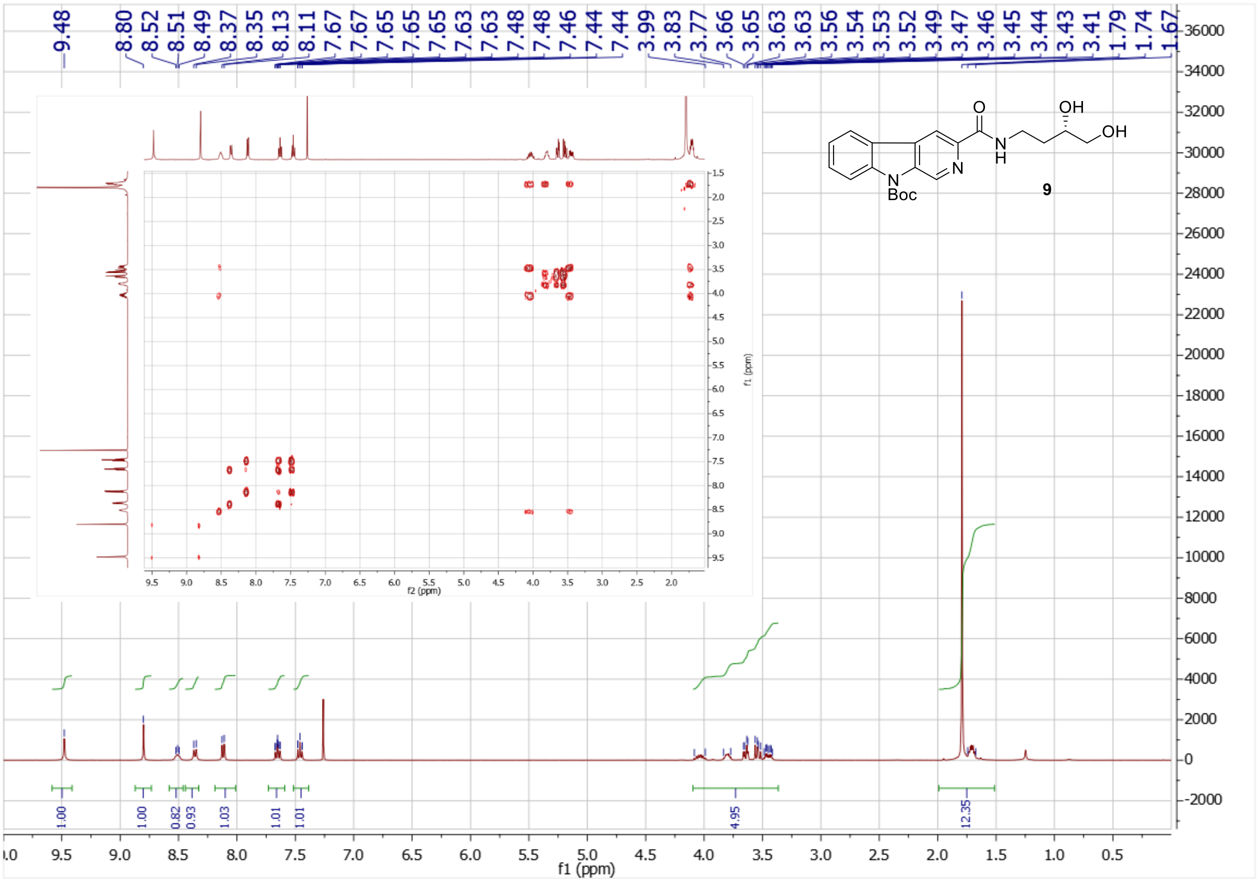

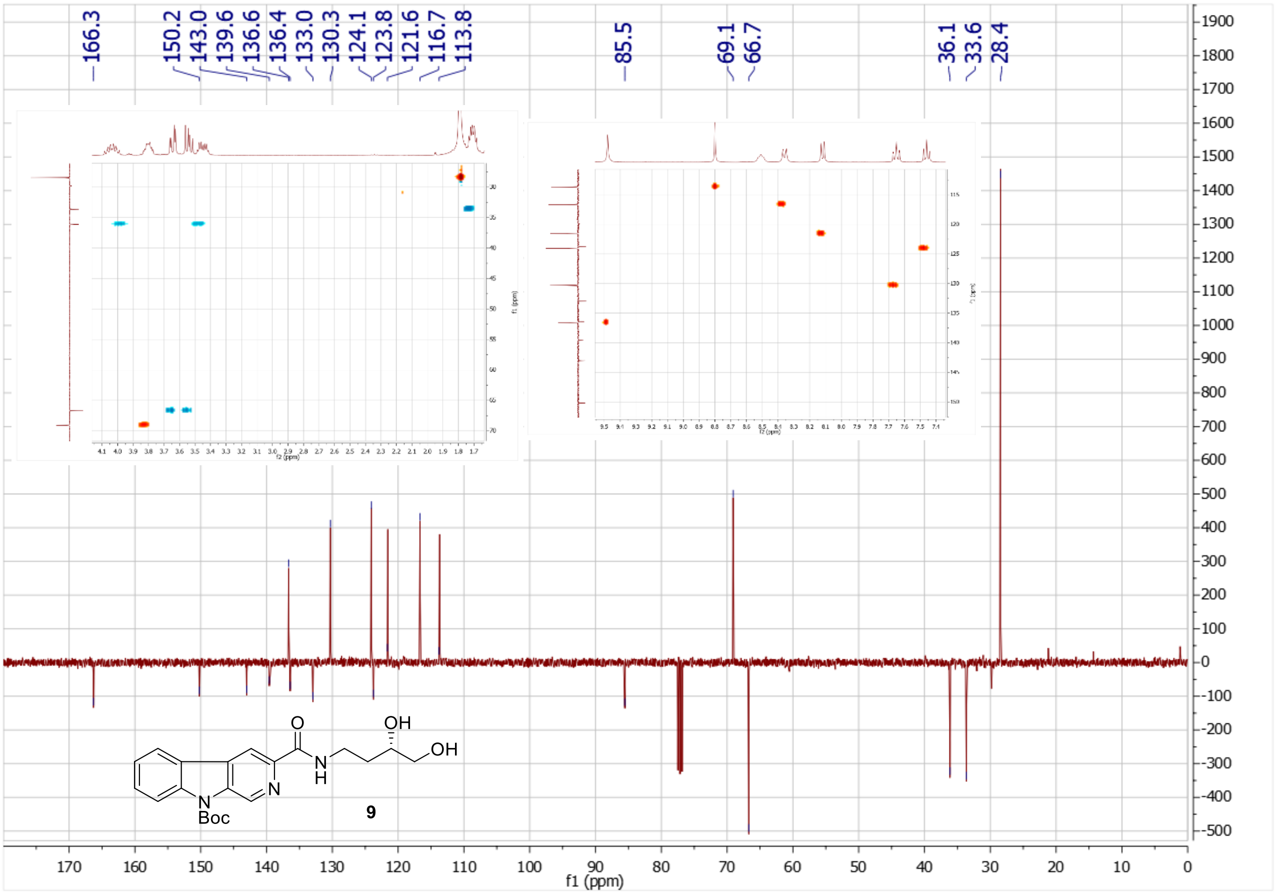

(9*H*-fluoren-9-yl)methyl tert-butyl (4-hydroxybutane-1,3-diyl)(*R*)-dicarbamate 14

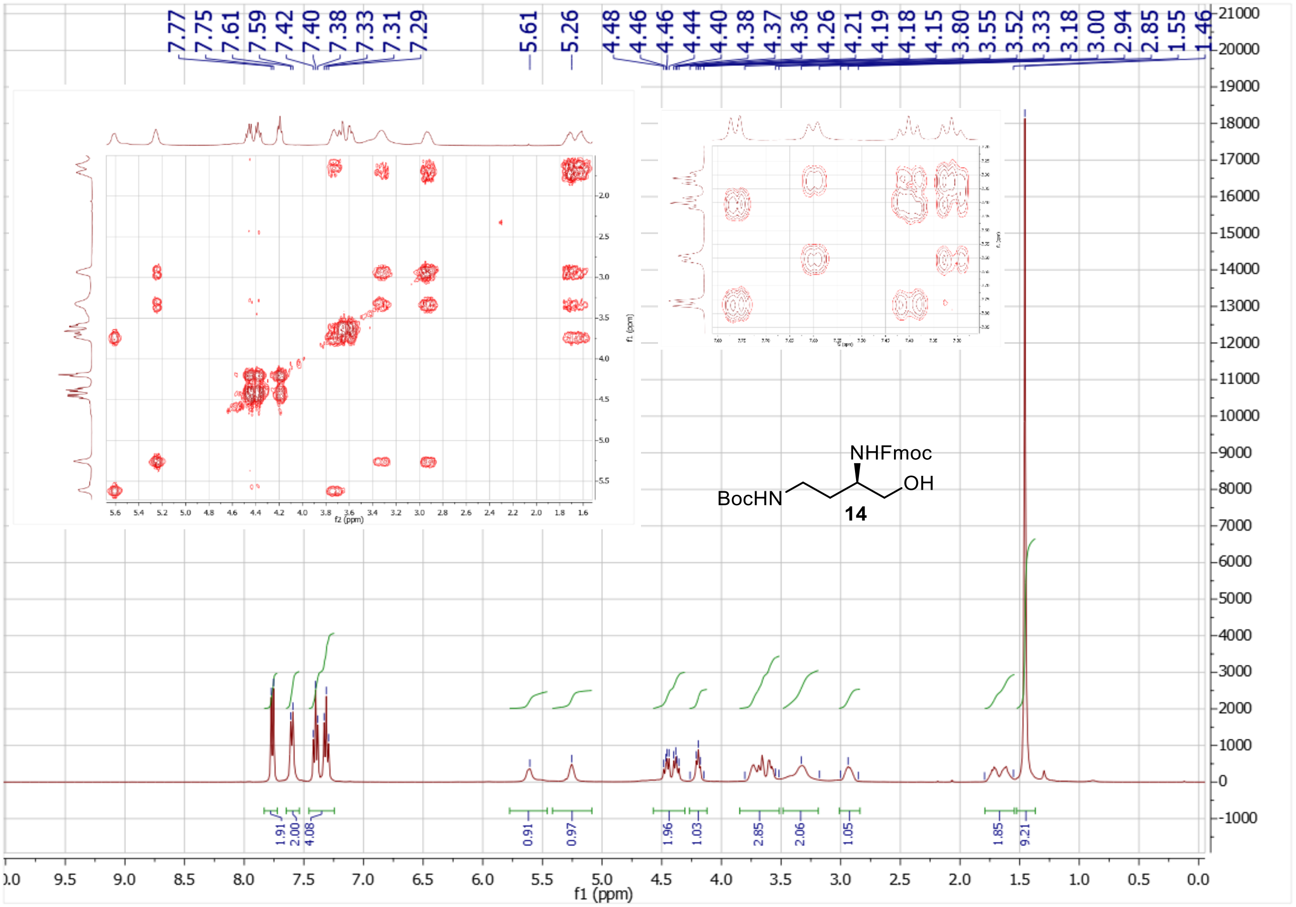

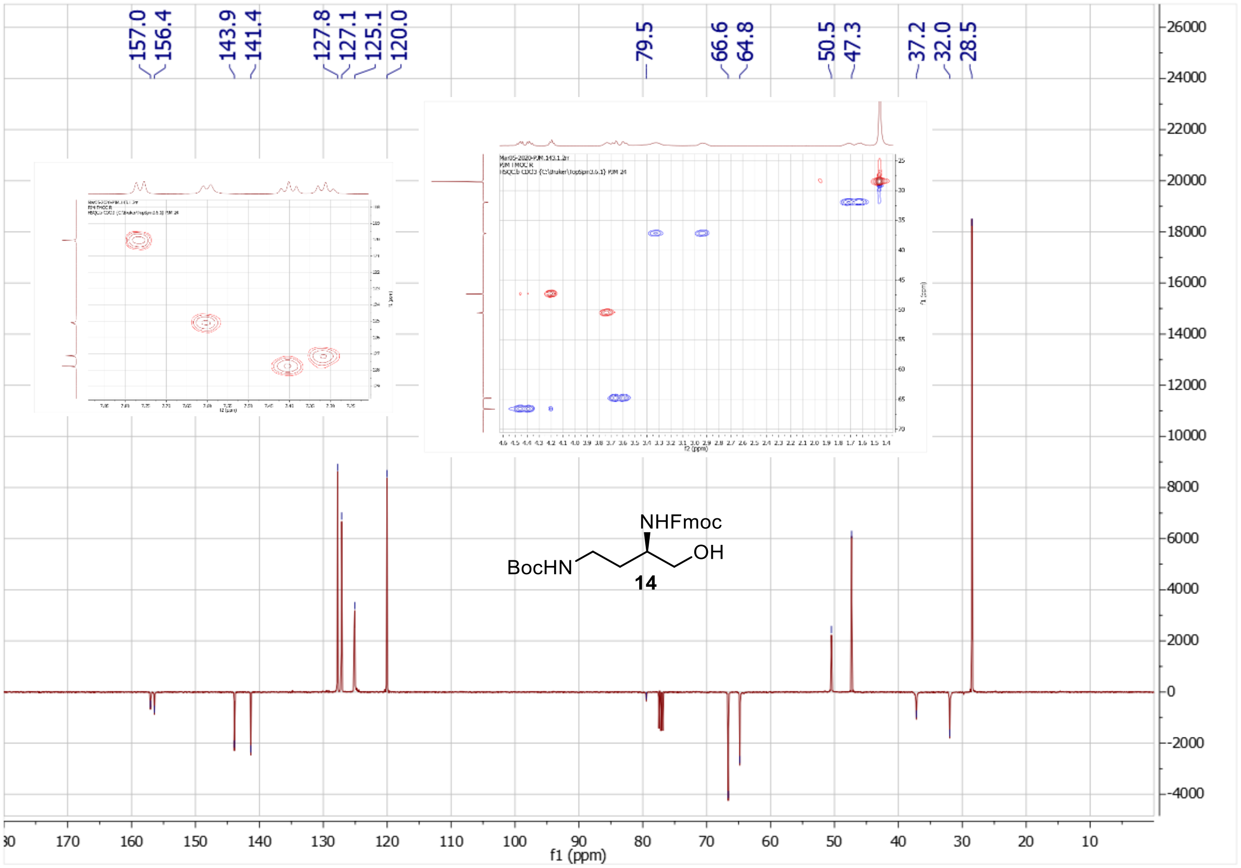

tert-Butyl _R_-(4-hydroxy-3-(_bis_-_tert_-butyl-guanidine)butyl)carbamate 16

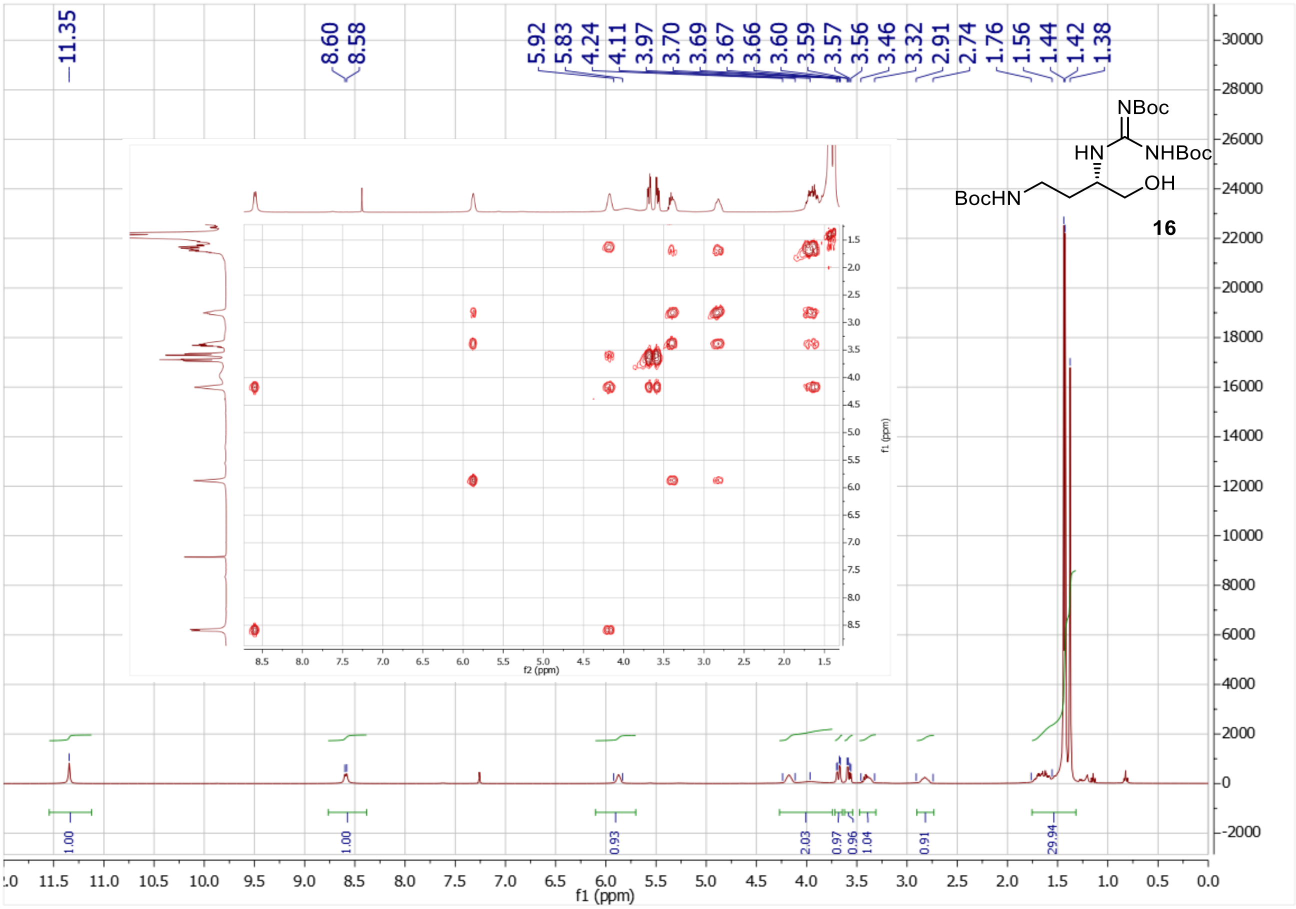

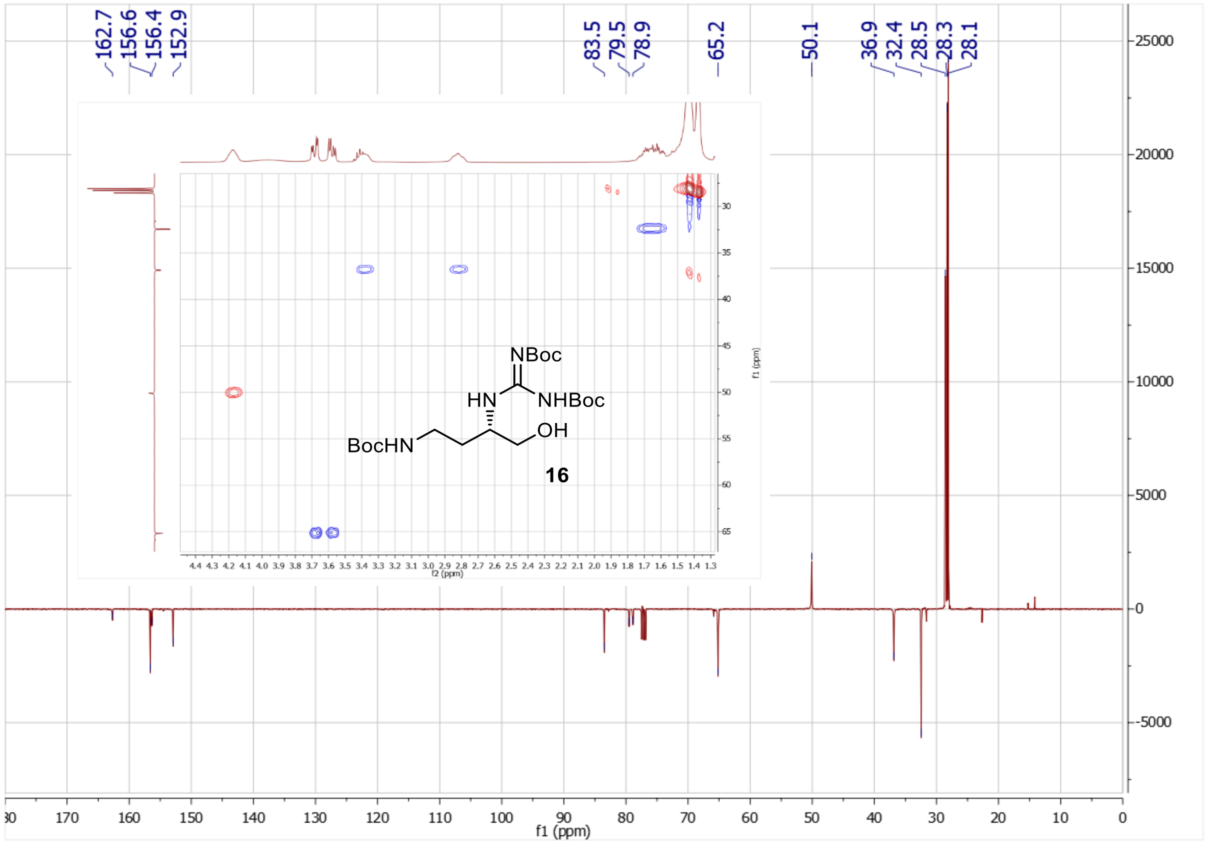

tert-Butyl (*R*)-4-(2-((tert-butoxycarbonyl)amino)ethyl)-2-((tert-butoxycarbonyl)imino)imidazolidine-1-carboxylate 17 (crude)

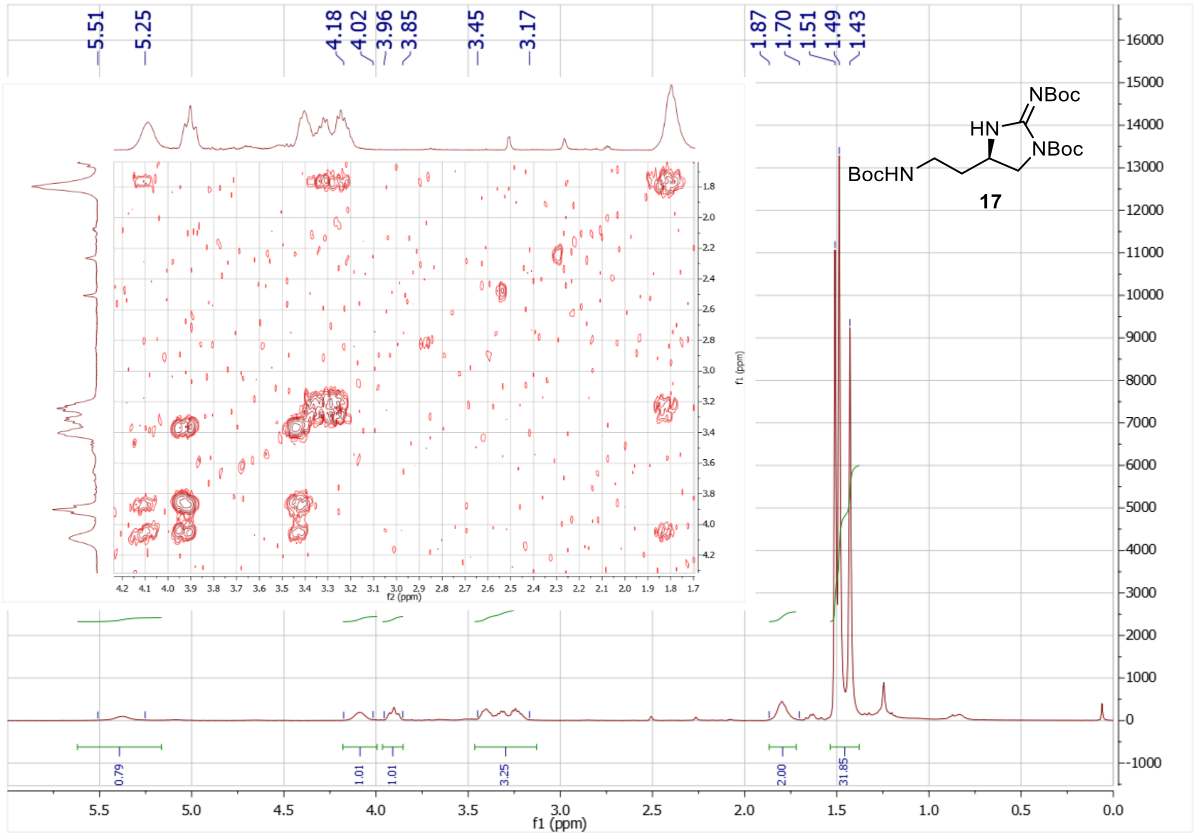

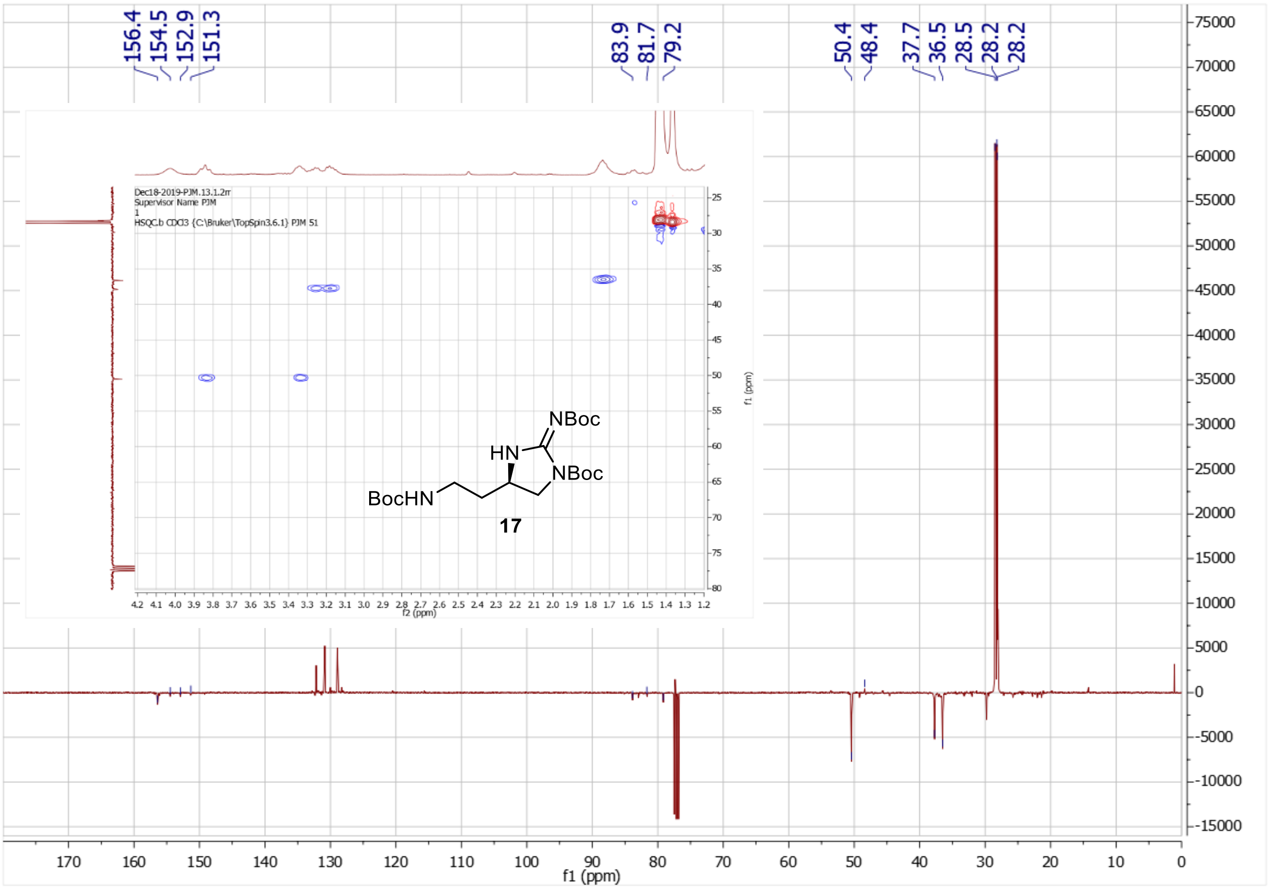

***R*-2-(2-iminioimidazolidin-4-yl)ethan-1-aminium dichloride *(R)*-5 (crude)**

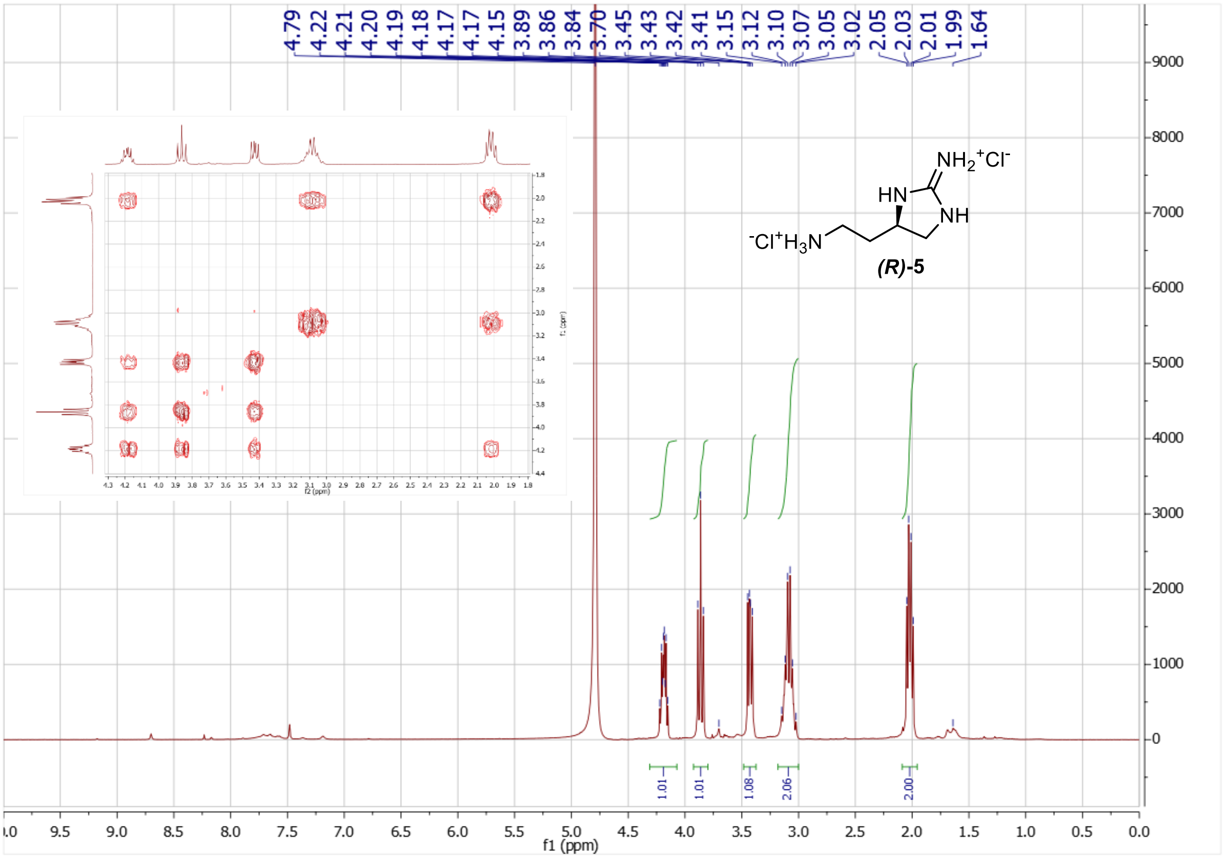

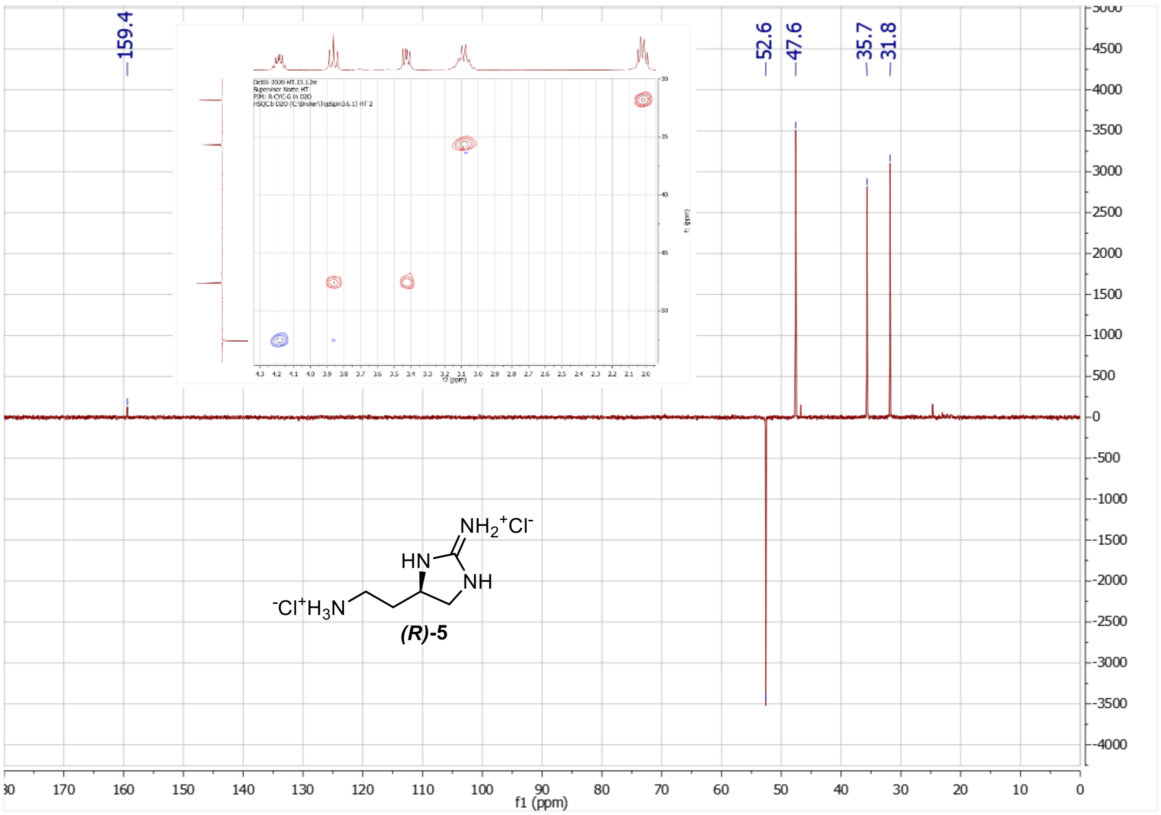

***(+)-(R)*-Tiruchanduramine 1·HCl.**

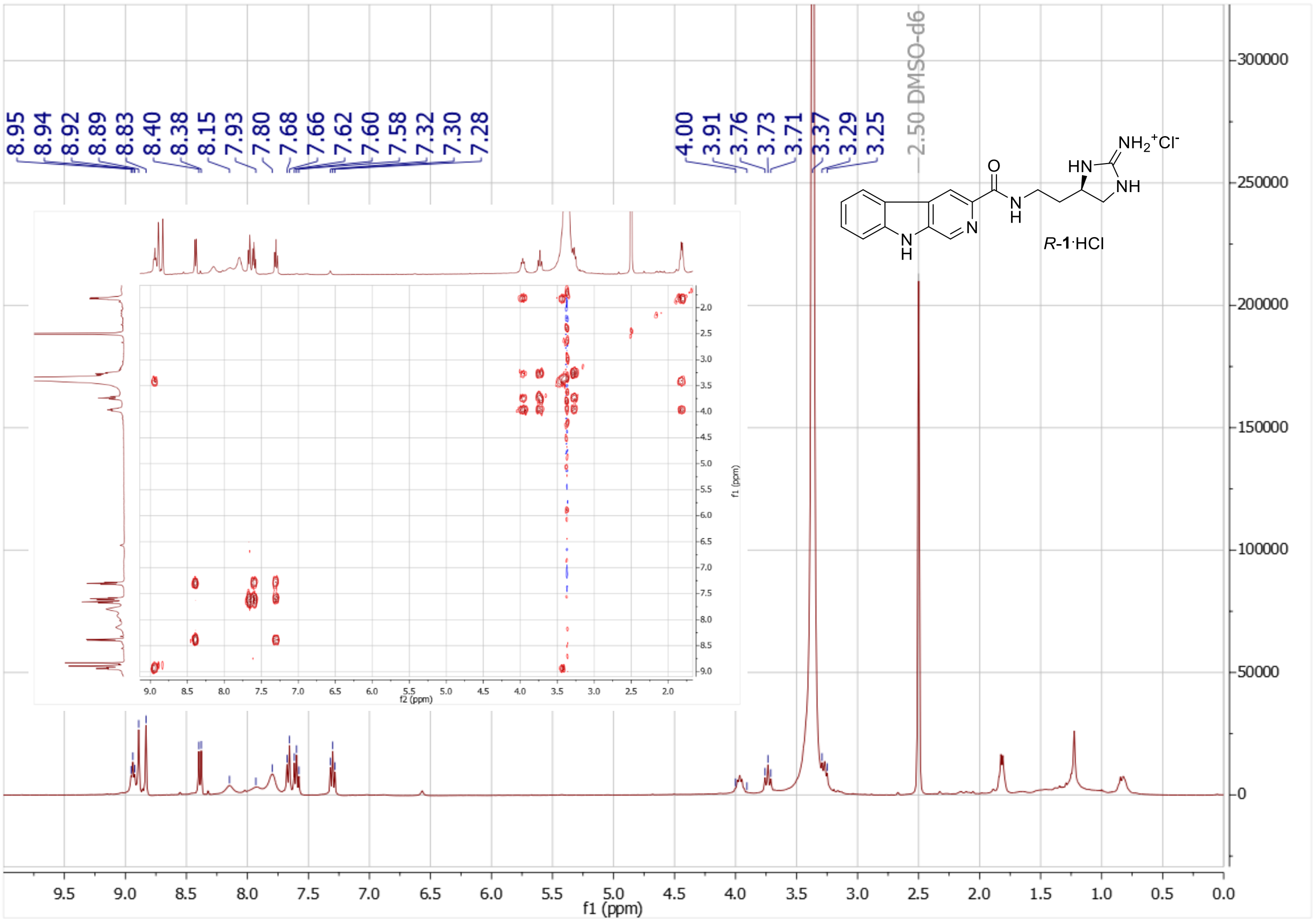

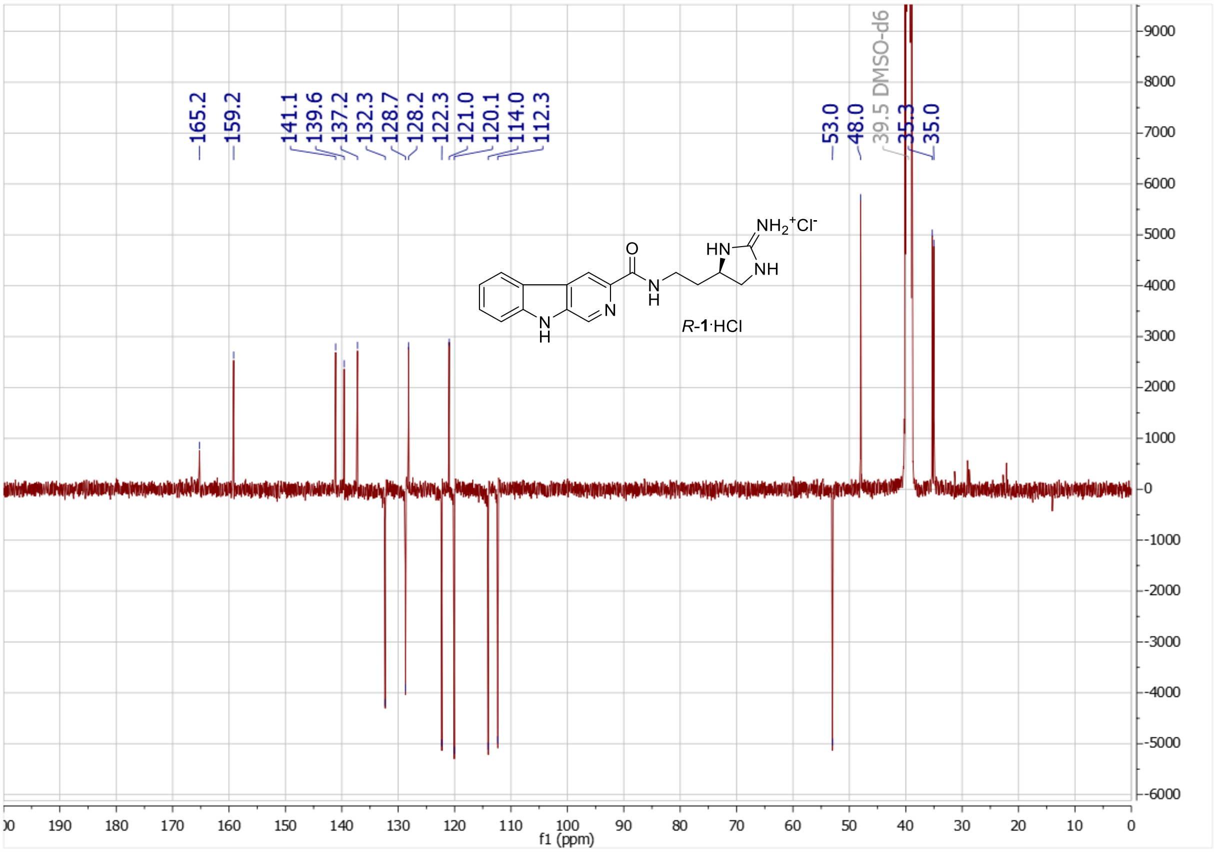

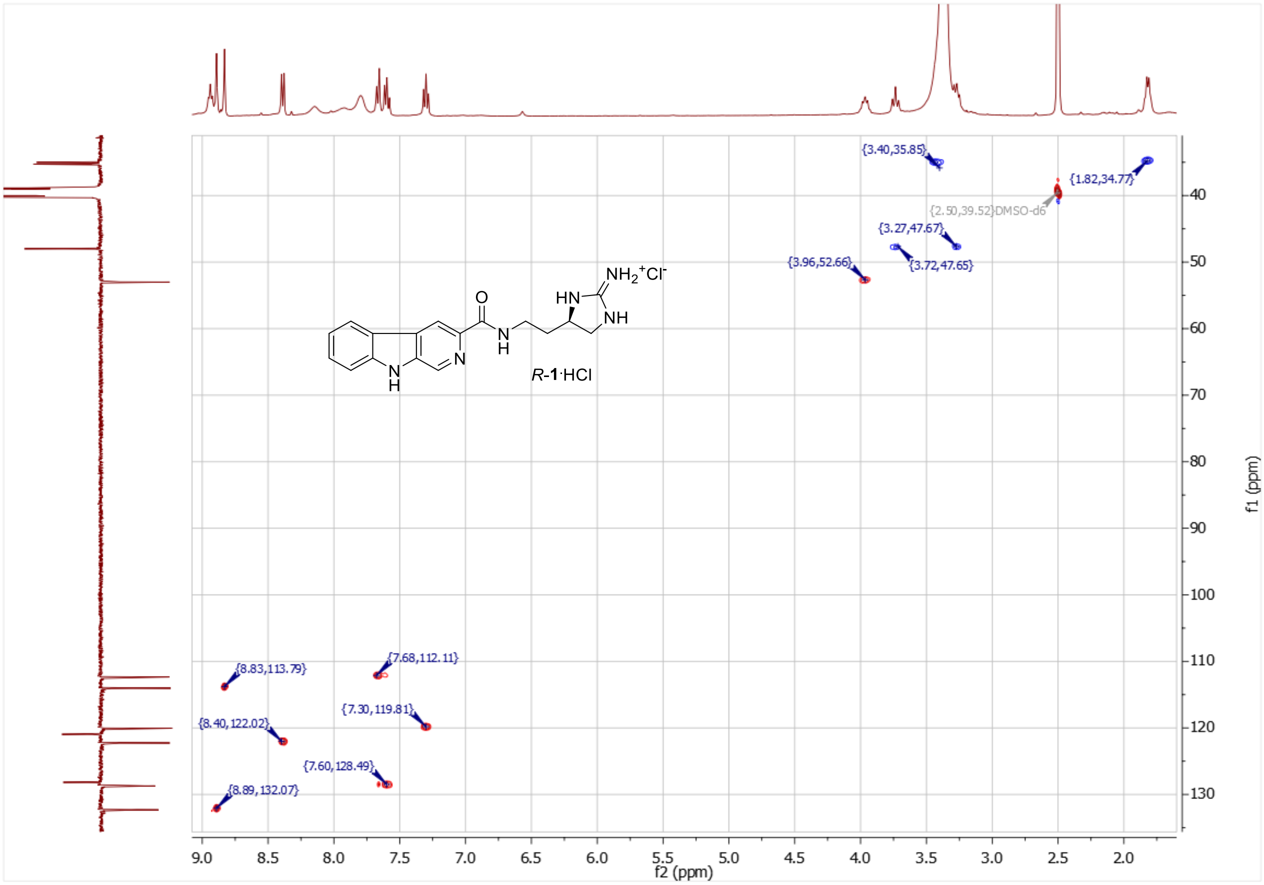

{3.40,35.85}
{1.82,34.77}
{2.50,39.52}DMSO-d6
{3.27,47.67}
{3.72,47.65}
{3.96,52.66}
NH2+Cl-
HN
NH
O
N
H
N
N
H
R-1·HCl
{8.83,113.79}
{7.68,112.11}
{7.30,119.81}
{8.40,122.02}
{7.60,128.49}
{8.89,132.07}
f2 (ppm)
f1 (ppm)

***(+)-(R)*-Tiruchanduramine 1·HCO₂H.**

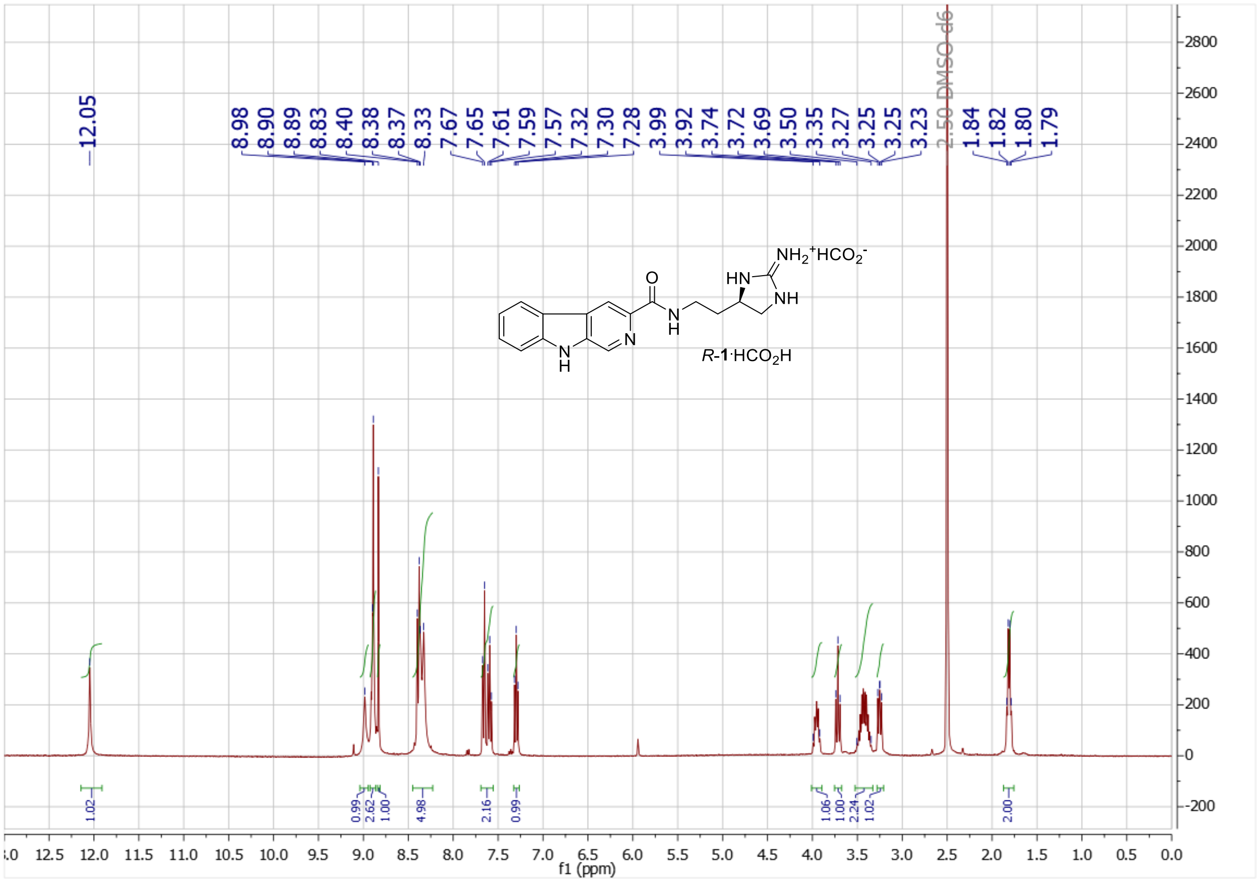

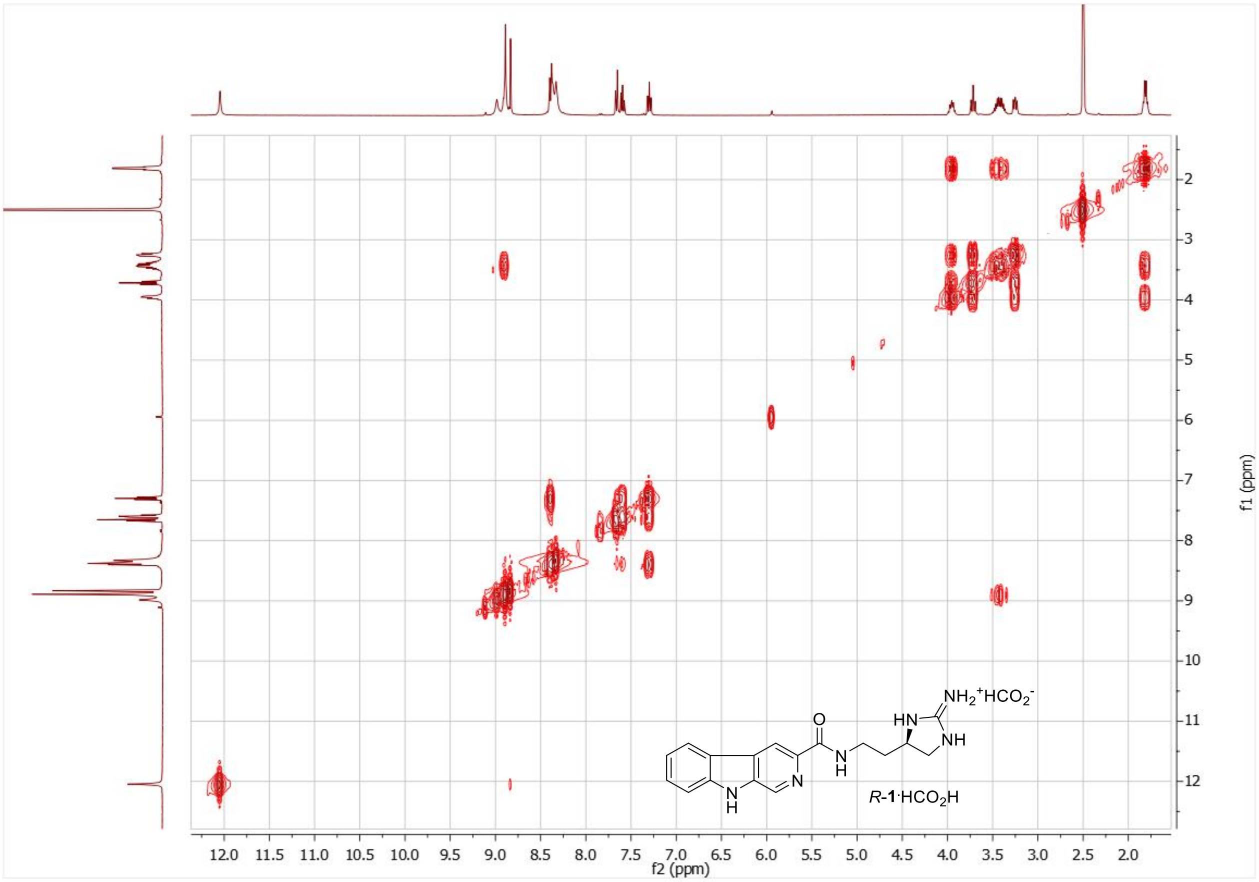

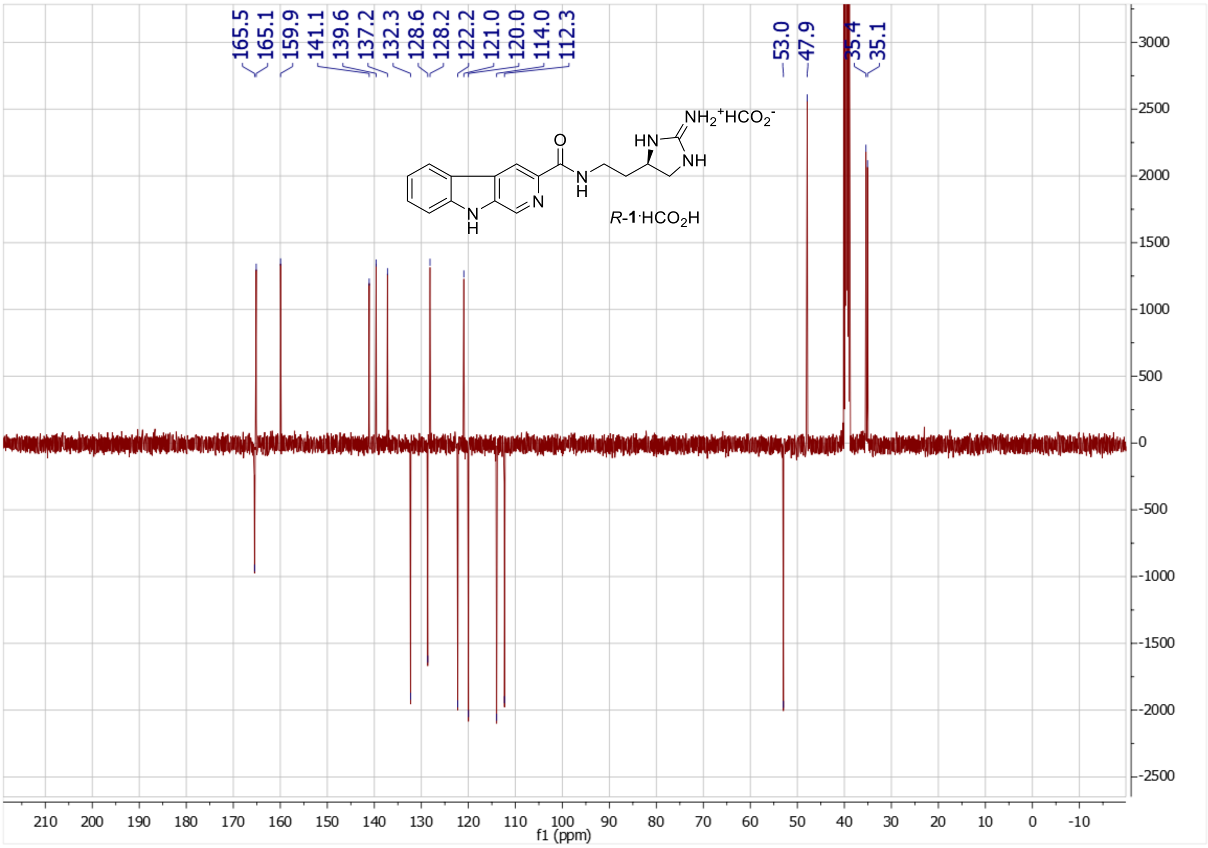
165.5
165.1
159.9
141.1
139.6
137.2
132.3
128.6
128.2
122.2
121.0
120.0
114.0
112.3
53.0
47.9
35.4
35.1
NH2+HCO2-
HN
NH
O
N
H
N
H
R-1·HCO2H
f1 (ppm)

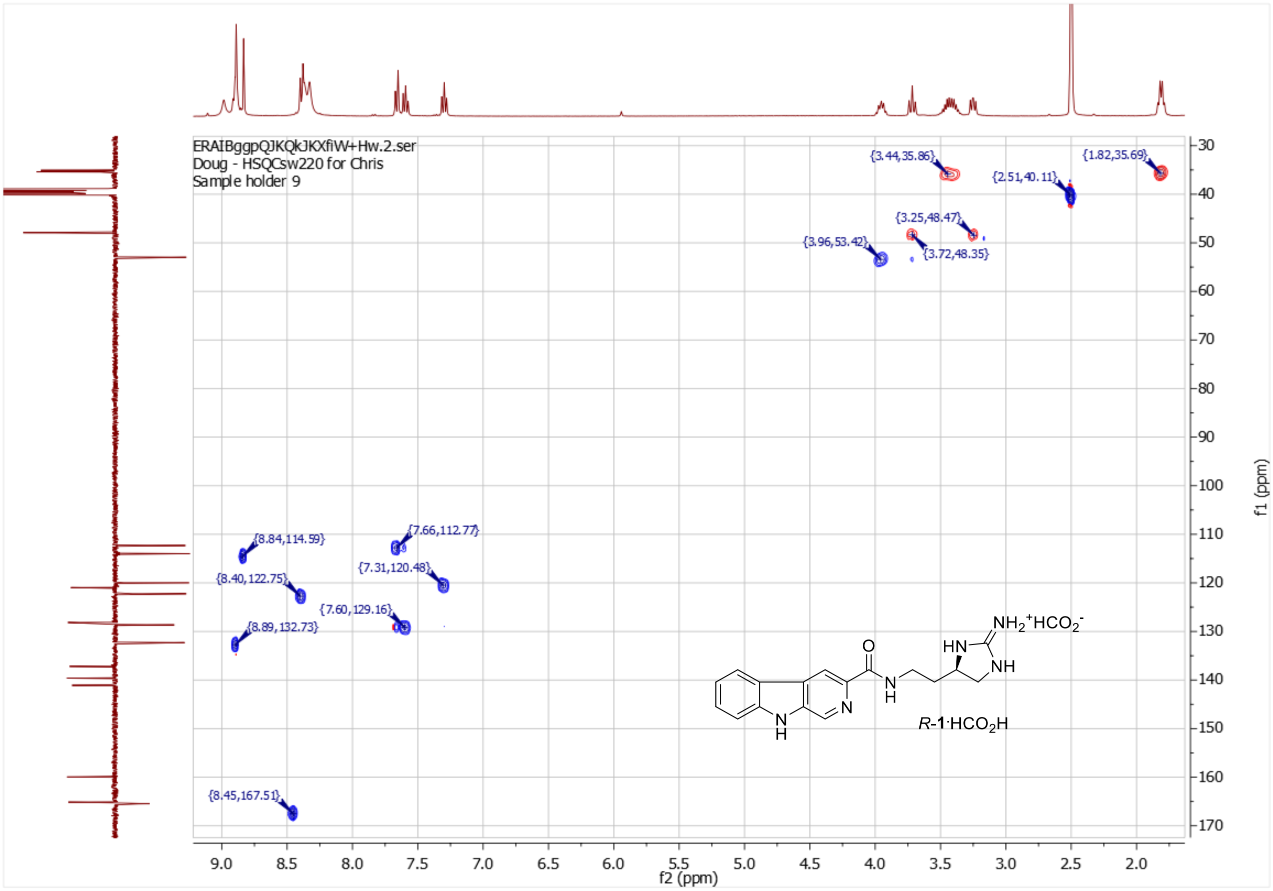
ERAIBggpQJKQkJKXfiW+Hw.2.ser
Doug - HSQCsw220 for Chris
Sample holder 9
{3.44,35.86}
{1.82,35.69}
{2.51,40.11}
{3.25,48.47}
{3.96,53.42}
{3.72,48.35}
{8.84,114.59}
{7.66,112.77}
{7.31,120.48}
{8.40,122.75}
{7.60,129.16}
{8.89,132.73}
{8.45,167.51}
NH2+HCO2-
HN
NH
O
N
H
N
H
R-1·HCO2H
f2 (ppm)
f1 (ppm)
